# How to engage across sectors: lessons from agriculture and nutrition in the Brazilian School Feeding Program

**DOI:** 10.1590/S1518-8787.2016050006506

**Published:** 2016-08-08

**Authors:** Corinna Hawkes, Bettina Gerken Brazil, Inês Rugani Ribeiro de Castro, Patricia Constante Jaime

**Affiliations:** I Centre for Food Policy. School of Arts & Social Sciences. City University London. London, UK; II Núcleo de Pesquisas Epidemiológicas em Nutrição e Saúde. Universidade de São Paulo. São Paulo, SP, Brasil; III Departamento de Nutrição Social. Instituto de Nutrição. Universidade Estadual do Rio de Janeiro. Rio de Janeiro, RJ, Brasil

**Keywords:** School Feeding, Sustainable Agriculture, Farmers Associations, Nutrition Programs and Policies, Food and Nutrition Security

## Abstract

**OBJECTIVE:**

To provide insights for nutrition and public health practitioners on how to engage with other sectors to achieve public health goals. Specifically, this study provides lessons from the example of integrating family farming and a nutrition into a legal framework in Brazil on how to successfully shift other sectors toward nutrition goals.

**METHODS:**

The study analyzed policy processes that led to a Brazilian law linking family farming with the National School Feeding Program. Main actors involved with the development of the law were interviewed and their narratives were analyzed using a well-established theoretical framework.

**RESULTS:**

The study provides five key lessons for promoting intersectorality. First, nutrition and health practitioners can afford to embrace bold ideas when working with other sectors. Second, they should engage with more powerful sectors (or subsectors) and position nutrition goals as providing solutions that meet the interests of these sector. Third is the need to focus on a common goal – which may not be explicitly nutrition-related – as the focus of the intersectoral action. Fourth, philosophical, political, and governance spaces are needed to bring together different sectors. Fifth, evidence on the success of the intersectoral approach increases the acceptance of the process.

**CONCLUSIONS:**

This study on policy processes shows how a convergence of factors enabled a link between family farming and school feeding in Brazil. It highlights that there are strategies to engage other sectors toward nutrition goals which provides benefits for all sectors involved.

## INTRODUCTION

In June 2009, a law was signed in Brazil requiring that 30.0% of the food budget of the national school feeding program should be used to purchase foods directly from family farms. Specifically, Article 14 of Law 11,947^a^ states that (except under specific circumstances):

At least 30.0% of the foods in school meals should be purchased directly from family farms and local rural enterprises, giving priority to the resettled farmers (former landless people), traditional indigenous communities and “quilombolas^b^” as a means of supporting local economic development.

By approving Article 14, Brazil became the first country to require by law a link between school feeding and agricultural production. The law represented the most recent in a series of changes to a national school feeding program – known in Brazil as *Programa Nacional de Alimentação Escolar* (PNAE – National School Feeding Program) – which has a history dating back to 1954. One of the very few programs in the world to be universal and free, PNAE served 45.6 million public school students in 2010 with a budget of 3.1 billion reais^c^. Its stated aim is “meeting the nutritional needs of students while at school, contributing to the growth, development, learning and academic achievement of students, and promoting the formation of healthy eating habits”^c^.

Article 14 was approved in the context of increasing international attention to the relationship between agriculture, nutrition, and health, including farm-school programs (or “home-grown school feeding”) around the world^2^ ^,^ ^8^ ^,^ ^14^, major research initiatives and events concerning agriculture for nutrition and health^13^, and numerous calls from the nutrition and public health community for the agricultural sector to play a greater role in reducing malnutrition and noncommunicable diseases^22^ ^,^ ^25^ ^,^ ^26^. At the same time, it is widely recognised that address malnutriton in all its forms will require inter-sectoral engagement.

Yet, successful collaboration between agriculture, nutrition, and health sectors has proved challenging^21^, with interests that may clash^6^. This also applies to inter-sectoral collaboration more broadly^15^. Thus, this study examined the example of integrating family farming and a nutrition into a legal framework in Brazil with the aim of identifying lessons on how to successfully shift other sectors toward nutrition goals. It focuses on the link between family farming and diet quality.

The study analyzed policy processes that led to the successful approval of Article 14. The precedent for this type of analysis is small – there are very few studies of policy processes in nutrition and health to date^1^. Yet the method is useful because it can “untangle the complex forces of power and process that underpin change”^4^ and help “identify strategies for increased political priority” for health^10^. The study of policy processes is also politically realistic: it assumes that policies develop because of an interaction between a whole host of actors and activities, values and interests, rather than simply as a direct result of the rational identification of a problem by the state^12^.

## METHODS

### Informant Interviews

Information and perspectives on the development of Article 14 were obtained from interviews with leading actors involved in the development of the law. The aim was to identify and interview the core set of actors involved in its development. Thus, an iterative methodology was used based on the perception of those actors. In the first instance, three people already known to the research team as having been involved in the development of Law 11,947 were contacted and asked to provide a list of people they perceived to be the main actors and entities involved. These actors were then contacted and asked the same question. The criteria for completing this process was that: (i) the same names, or names of organizations, appeared in most lists; (ii) the main entities – which emerged as politicians; government ministries; the *Conselho Nacional de Segurança Alimentar e Nutricional* (CONSEA –National Council on Food and Nutrition Security); family and peasant farming groups; and food security civil society groups – were represented. The results were very consistent, leading to a list of 17 names. A recognized limitation of this process was that it was based on the perception of actors who had collectively supported the law. It was natural that actors would not name opponents of the law as having been actively involved in its development, (since they tried to block it), nor those unknown to them who may have wanted to be more involved in its development. To provide a more independent view, two academics with expertise on the PNAE were added to the list, making a list of 19 names*.*

A total of 18 people were interviewed, following an interview protocol, during February and July 2010 (one person on the list of 19 was unavailable for interview). The questions in the protocol were developed with the aim of uncovering the nature of the intersectoral approach, interests and values involved, and factors both facilitating and presenting barriers to the approval of Article 14. The protocol was adapted according to the need of each key informant. Interviews were conducted separately for each key informant in person or by phone with two members of the research team; in one case, the respondent answered via e-mail. All the interviews except three were conducted in Portuguese, the others being conducted in English. The interviews lasted between one and four hours. Four of the informants were interviewed twice. The oral interviews were recorded and transcribed and translated into English.

As a study of political processes in the field of Social and Human Sciences, the assessment of ethical aspects does not apply, according to CNS Resolution 466, of December 12, 2012. Interviewers were informed of the relevance of the study and the ethical issues involved.

In addition to the interviews, information was sought from documents on the history of the development of the law, and key antecedent actions, from published documents.

### Analysis

The analysis of the content of the interviews took a thematic approach based on a well-established theoretical framework of policy process^12^. The model aims to explain why issues and problems become prominent in the policy agenda and are eventually translated into concrete policies. It theorizes that policies are made when what are termed different “streams” converge at a crucial moment to open a policy window: the “problem stream” (the problems that the policy is concerned with addressing); the “policy solution stream” (the ideas that emerge to solve the problems); and the “political stream” (the developments in the political context). An important notion in the model is that proposals for solutions in the policy stream are not necessarily built to resolve given problems; rather, they float in search of problems to which they become tied when, in “policy window” events, when advocates are able to push their favored policies into the political agenda.

The framework used in this study also incorporates another widely used concept in the study of policy processes: that of an “advocacy coalition”^12^. In this theory, advocacy coalitions – groupings of policy actors with a shared set of values and beliefs who advocate policies that align with their values – are a crucial component of policy change.

This narrative of the interviews, which proved fairly consistent between the informants, was then organized and analyzed according to these themes: problems, policy solutions, and politics; and then used to identify key lessons learned.

## RESULTS AND DISCUSSION

The study provided five key lessons for promoting intersectorality, based on the Brazilian experience of linking family farming with the National School Feeding Program, namely that enabling intersectorality for nutrition requires: 1) a triage of political, philosophical, and governance spaces to bring together different sectors; 2) coalitions with more powerful sectors focused on achieving a common goal; 3) positioning nutrition goals as a solution that meets the interests of other sectors; 4) obtaining evidence that the intersectoral approach can work; 5) not being afraid of bold ideas. We now present the findings of the study which led to the identification of these five lessons.

### Lesson One: Identify or Create a Triage of Spaces to Bring Together Different Sectors – Political, Philosophical, and Governance Spaces

Article 14 emerged in the context of several perceived problems. The two most important problems were that of food insecurity and the challenges faced in Brazil by family farmers.

Current approaches addressing food security among low-income Brazilians and vulnerable groups, such as indigenous people and *quilombolas*, refer to the end of Dictatorship in 1984, with the emergence of an active civil movement^16^. It was not until 2002-2003, however, that food security moved to the center of the political stage with the election of the Workers Party. Ending hunger had been a core election pledge of the new President, Luiz Inacio Lula da Silva (“President Lula”). He also had a program to do so: *Fome Zero* ^16^ ^,^ ^d^. The program emphasized the need for structural reform to address income poverty and encourage the production of lower cost food, including by supporting family farmers, as well as the expansion of specific programs. Notably, the government established its cash transfer program, *Bolsa Família*, in 2003 to provide financial aid to poor Brazilian families.

Another of the initial steps taken to implement *Fome Zero* was the reestablishment of CONSEA in 2003^3^ ^,^ ^e^. A large cross-sectoral government-civil society group established to advise the President on policies and actions needed to promote food and nutrition security; it is with CONSEA that the story of Article 14 starts^f^. As part of the process of developing a work plan, the president of CONSEA held discussions with various government bodies with food security concerns, including the *Fundo Nacional de Desenvolvimento da Educação* (FNDE – National Fund for Educational Development), a body of the Brazilian Ministry of Education that manages the PNAE (Table). The FNDE reported that their key issue was resources: anecdotal evidence and research^18^ had shown that municipalities have insufficient resources to consistently provide school meals of sufficient quality, and the PNAE management wanted more per capita expenditure per student.

**Table:** Key actors and their roles and responsibilitiesa.

Actor	Core functions	Specific subactors	Role and responsibilities in developing Article 14
Intersectoral between government and civil society			
*Conselho Nacional de Segurança Alimentar e Nutricional* (CONSEA – National Council on Food and Nutrition Security)	Cross government-civil society advisory group to the president on policies and actions needed to promote food and nutrition security.	Working group for School Feeding CONSEA presidents	- Drafting the law; - Placing the issue on the agenda of government ministries; - Lobbying congress (arranging meetings etc.); - Providing technical expertise; - Providing the space for intersectoral discussions.
Government^b^ - ministries and agencies			
*Fundo Nacional de Desenvolvimento da Educação* (FNDE – National Fund for Educational Development) of the Brazilian Ministry of Education	Government agency responsible for managing the *Programa Nacional de Alimentação e Nutrição* (PNAE – National School Feeding Program).	Coordinator of the PNAE *Coordenação Técnica de Alimentação e Nutrição* (COTAN – Technical Coordination on Food and Nutrition) composed of nutritionists of the FNDE and external experts	- Contribution in drafting the law as a member of the CONSEA working group; - Coordinating the discussion between the FNDE, the Brazilian Ministry of Education, CONSEA, and the Congress; - Redrafting the bill into a Provisional Measure (MP).
Brazilian Ministry of Agrarian Development (MDA)	Government ministry responsible for developing and implementing policies for family farming.	Division of income generation and value addition of *Secretaria da Agricultura Familiar* (SAF – Secretariat of Family Farming), responsible for developing and testing innovative policies and programs to increase market access for family farmers and the market value of their products.	- Lobbying politicians to support the law; - Providing evidence on the workability of PAA; - Member of CONSEA.
*Companhia Nacional de Abastecimento* (CONAB – National Food Supply Company	Public company attached to the Brazilian Ministry of Agriculture, Livestock and Food Supply responsible for controlling the supply and trade of food stocks, acquisition of stocks from farmers and setting prices, including setting minimum prices for the *Programa de Aquisição de Alimentos* (PAA – Food Acquisition Program).	*Diretoria de Política Agrícola e Informações* (DIPAI – Board of Agricultural Policy and Information)	- Convincing other government officials that buying from family farmers was feasible and desirable; - Providing evidence on the role of PAA; - Member of CONSEA.
Government – politicians			
*Frente Parlamentar de Segurança Alimentar e Nutricional* (Parliamentary Front on Nutrition and Food Security), led by Deputy Nazareno Fontales	Group of 250 parliamentarians from the Chamber of Deputies and the Senate advocating and articulating laws that aim to improve food and nutrition security.	Directed by Deputy Nazareno Fontales	- Ensuring the technical approval of the law by guaranteeing its structures were in place in the Congress; - Advocating the law in the Congress; - Negotiating the text of the law to build consensus among the Congress; - Proposing amendments that allowed the law to be approved; - Informing CONSEA whom in the Congress needed lobbying, and when - Conducting dialogue with CONSEA and civil society.
President Luiz Inacio Lula da Silva	President of Brazil.		- Requesting CONSEA to develop ideas to improve the PNAE; - Exhibiting high-level support for the law.
Senator for the state of Rio de Janeiro, Francisco Dornelles	Senator and rapporteur for the bill.		- Lead opponent of Article 14 and the law.
Civil society – food security			
*Fórum Brasileiro de Soberania e Segurança Alimentar* (FBSSAN – Brazilian Forum on Food and Nutrition Sovereignty and Security)	Network of NGOs aiming at communicating the importance of food security among society as a whole and encouraging the development of public policies to improve food security.	- Current president of the NGO FASE; - State level forums.	- Mobilizing and building support from different sectors (drafting letters, disseminating information, etc); - Hosting a national seminar (70 people) in 2008; - Creating an intersectoral committee from civil society to monitor the progress of the law; - Lobbying congress (e.g., leaflets, organizing e-mail campaign to congressmen; meetings with the Parliamentary Front; hearings with party leaders).
*Federação de Órgãos para Assistência Social e Educacional* (FASE – Federation of Organizations for Social and Educational Assistance)	NGO concerned with food security.	- Member who was p0resident of FBSSAN.	- Member was the president of FBSSAN; - Member of CONSEA.
*Ação Brasileira pela Nutrição e Direitos Humanos* (ABRANDH – Brazilian Action for Nutrition and the Right to Food)	NGO concerned with the right to food.	Director.	- Involvement with FBSSAN work (see above); - Meeting with members of the Congress; - Mobilizing support; - Member of CONSEA.
Civil society - family farmer groups			
*Confederação Nacional dos Trabalhadores na Agricultura* (CONTAG – National Confederation of Workers in Agriculture)	Labor union of rural workers.	- Women’s Bureau of CONTAG.	- Mobilizing their members; - Lobbying the Congress; - Coordinating with CONSEA, FNDE.
*Federação dos Trabalhadores na Agricultura Familiar* (FETRAF – Federation of Workers in Family Farming)	Labor union of workers for family farms.		- Mobilizing their members; - Lobbying the Congress.
Via Campesina	Network of NGOs concerned with food sovereignty, land reform, and peasant farming.	Movement of Women Peasants; Landless Rural Workers’ Movement (MST); Movement of Small Farmers (MPA).	- Mobilizing their members; - Lobbying the Congress.
*Articulação Nacional de Agroecologia* (ANA – National Network of Agroecology)	Network of NGOs that promotes agroecological methods in farming.	Head of working group on food security.	- Member of CONSEA; - Mobilizing support from members.

^a^ The study did not include interviews or analysis of the role of state level actors. However, several informants noted that actions were also taken at state level. This included the CONSEA that existed at state level – which advocated for Article 14 –, the state forum FBSSAN, the ANA – which advocated their state deputies and senators in favor of the law –, and municipal mayors and secretaries of education, some of whom had doubts about Article 14.

^b^ The Brazilian state apparatus is structured in three branches: executive (ministries, agencies etc.), legislative (an elected Senate and Chamber of Federal Deputies, termed together by the Congress), and judiciary. It has a constitution originally ratified in 1988.

The president of CONSEA subsequently discussed the issue with President Lula, who was reported to say that he was committed to a gradual increase in expenditure – and also requested that CONSEA prepare some suggestions about how to improve PNAE more comprehensively. Then, CONSEA made school feeding one of their core strategic priorities and established a working group to develop ideas. At first, there was no discussion about developing a new law, but the working group identified many failings in the existing PNAE program due to lack of what they termed an “institutional framework”. Therefore, they started drafting a bill, completing the first draft in the first half of 2006, including an extension to all students engaged in basic education in the public system, a framework for increasing the *per capita* expenditure per student, a requirement that nutritionists design the menus, a ban on outsourcing to private meal contractors, and what was to become Article 14.

The idea behind Article 14 was already very familiar to the CONSEA membership^g^. As said by one member of CONSEA, it was “an idea with a history”^h^. Part of that history was a second “problem”: the challenges faced by family farmers. The concept of “family farmers” emerged sometime in the 1980s^17^. In 1995, as part of the agricultural market liberalization program of President Fernando Henrique Cardoso, the government established the *Programa Nacional de Fortalecimento da Agricultura Familiar* (PRONAF – National Program to Strengthen Family Agriculture), which focused on providing credit to family farmers. However, rather than perceiving family farming as an economically dynamic category of its own, PRONAF aimed to help integrate family farmers into the agribusiness chain^20^.

The perception of family farmers began to change in 2000, when an international report produced data revealing the importance of family farmers to food security in Brazil.^9^ Family farmers were found to be responsible for ensuring much of the country’s food security as a major food supplier to the domestic market of products core to the Brazilian diet (e.g., cassava, milk, corn, rice, pork, and poultry). Evidence also showed that family farming generated around one-third of agricultural revenues and represents over 85.0% of all rural businesses (around 4.1 million farms), but receives just 25.8% of financing targeted at agriculture. Although almost 50.0% of family farmers had no access to assets or resources and were very poor and vulnerable to food insecurity, resettled family farmers generated better incomes than wage labourers^9^ ^,^ ^11^. Thereupon, this stimulated a series of developments aimed at supporting family farmers. For example: in 2001, a ministry dedicated to family farming was established, the *Ministério do Desenvolvimento Agrário* (MDA – Brazilian Ministry of Agrarian Development)^i^; in 2003, the new government of President Lula created the *Programa de Aquisição de Alimentos* (PAA – Food Acquisition Program), which purchases food directly from family farms and distributes it to institutions and families at risk of food and nutritional insecurity by social programs^7^ ^,^ ^j^; in 2006, the term “family farming” was defined in law (farms with less than a specified number of “fiscal modules” operated by the owner with predominantly family labor)^k^ .

Members of CONSEA were very familiar with the contribution made by family farmers to national food security and recognized that many remained poor. They were also very familiar with PNAE. As such, they were able to put both together. According to one leading food security activist:

When we bring this perspective of food and nutrition security, we begin to make some articulations and ask some questions. First, we know that family farming is the main supplier of our domestic food, we know it is a segment that has an economic and social role that needs to be recognized, we know that some family farmers are food insecure. The second is that PNAE is one of our most important programs for food security. And the amount of food that is purchased by this program is fantastically large. So we ask: where does the food come from? Well, it could come from family farmers! It is just a question of joining both sides. Profit on both sides! In this framework, it is easy to understand why we have made a link between family farming and institutional markets.

As indicated in the quote, it was not just food security that was the concern of social actors, but also nutrition security. The addition of nutrition into the concept of food security was forged during the process, and formally approved, at the second *Conferência Nacional de Segurança Alimentar e Nutricional* (CNSAN – National Conference on Food and Nutrition Security), in 2004^l^. According to this broader concept, public policies on food and nutrition security, as a way of ensuring the human right to adequate food, should not only encompass actions to improve availability of and access to food, but also promote and protect sustainable and healthy diets. This concept links the nutritional dimension of food security that puts all sectors, their priorities, and their agendas in the same space. The crucial here, then, was this suprasectoralframework of food and nutrition security, the philosophical space, which enabled the two problems to be placed together^3^.

Moreover, food and nutrition security had been given a policy space in Brazil (in the form of *Fome Zero*) with high political legitimacy. And crucially, CONSEA provided the governance space to make it happen – the “intersectoral space to have the conversation” between “the three main actors – government, managers of the school feeding program, and social movements”. As such, CONSEA was consistently cited by informants as the “main actor” and “central” to the approval of the law. It “gathered people who think about food security in Brazil”, said one, who were “also concerned about healthy eating” and had the “conviction to allow the construction of the bill”.

### Lesson Two: Forming Coalitions with More Powerful Sectors Focused on Achieving a Common Political Goal that Can Help Moving Them Toward Nutrition and Health Goals

After the draft bill containing Article 14 had been accepted by the CONSEA membership, it was analyzed by *Casa Civil* in 2007^m^. At the end of this process, the bill was approved by the president, who presented it to the Congress in 2008. The bill was approved in full by the Chamber of Deputies, and then forwarded to the Senate – but not approved. The senator whom had been appointed to be the rapporteur was vigorously opposed to the whole concept of the Bill and recommended it not to be approved unless Article 14 was removed, along with the ban on the use of outsourcing.

What followed was fierce lobbying in favor of Article 14 and the bill as a whole – a process strengthened by the presence of three overlapping advocacy coalitions. The first was between the relevant government ministries and agencies who had first discussed family farming-PNAE links as members of the Executive Steering Group of an existing government program (see Lesson 4)^n^. According to a key informant from FNDE, this coalition was essential in ensuring the approval of the bill in the political process, particularly the juridical one, because it meant that “the FNDE was not alone”.

The second coalition was formed by food security advocates. This coalition had begun to emerge in the 1990s with the founding of *Fórum Brasileiro de Soberania e Segurança Alimentar* (FBSSAN – Brazilian Forum on Food and Nutrition Sovereignty and Security), and was politically consolidated in the campaign in which *Lei Orgânica de Segurança Alimentar e Nutricional* (LOSAN – Law of Food and Nutrition Security) was approved, in 2006. The experience of developing LOSAN led a politician who had been much involved to found and lead the *Frente Parlamentar de Segurança Alimentar e Nutricional* (Parliamentary Front on Food and Nutrition Security) of over 230 deputies and senators in 2007^o^. The Parliamentary Front, in turn, partnered with civil society to advocate their cause and were said to be “crucial to the process”.

The third coalition was of family farming advocates. Almost all the informants noted the importance of the strong mobilization and extensive, well-organized advocacy activities by the coalition of family farming groups. Such as the advocacy coalition around food security, the coalition included those outside the government – from worker unions to peasant groups – and inside it (the MDA, for instance, etc.) as “the institutional framework for dialogue with family farming movements”.

During this time, several changes were made in the bill: the removal of the article banning outsourcing; the introduction of “exceptions” to Article 14 meaning that in specified situations it was illegally required to apply it (and the later deletion of one of these exceptions); and the addition of the term “directly” to Article 14 to clarify that the intention was that municipalities should purchase directly from farmers, rather than via a third party^p^. Following these changes and the strong political pressure for the law to be approved, the senator “retreated” from his previous position and the law was unanimously approved in the Congress. It was then sent again to *Casa Civil* for a final legal review and, in June 2009, signed into law by the president.

The presence of a strong advocacy coalition was clearly highly significant in the approval of the bill. However, the process lacked a clearly distinguishable coalition formed around nutrition and health interests. That is not to say the nutrition community was absent: nutritionists were present in the PNAE coordinating unit and their technical subgroup on food and nutrition, *Coordenação Técnica de Alimentação e Nutrição* (COTAN – Technical Coordination on Food and Nutrition); in food security committees; and formed the largest proportion of the 15-strong CONSEA working group on school meals. These nutritionists were concerned about the quality of schools meals: it was reported that a survey of menus by COTAN, in 2006, 41.0% of meals did not contain any fruit in any week, and 16.0% had no vegetables^q^. A 2004 study of 1,000 schools also found fruit and vegetable servings inadequate, while artificial juices were widespread (in 59.0% of schools).

But these concerns were reportedly certainly not central to the discussions. The deputy leader of the Parliamentary Front on Food and Nutrition Security was, according to one of the officials in his office, very concerned about health. As a medical doctor, he believed that healthy eating was central as a means to “promote healthier nutrition and prevent diseases such as obesity…you can’t have on one side the fight against hunger, but then let the other side of obesity replace it”. However, according to this deputy, “though I mentioned it in the discussion, the issue of health was quite unvalued. During the discussion with Chamber of Deputies, there was not much emphasis on children’s health.” Another informant also said “there was no interest in healthy eating inside the Congress.”

The family farming interests were also reported to not speak much about health. “Discussing eating habits is a novelty for farmers”, said one key informant; “the concept of healthy eating was more important to the food security people than to the family farmers”, said another. “Healthy food” was indeed important for the food security advocates involved, but came into the discussions “as if by osmosis”. The key discussion point was not the nutritional standards of school meals – which was never mentioned by anyone – but replacing the foods of the past – the “cookies from Sao Paulo” – with fresh “basic foods”. As put by one member of CONSEA, “what was being served was not healthy food, and we believe that children should receive a proper meal in schools, not just a snack or re-hydrated food”. And it family farmers were believed to best provide these “basic” foods.

Likewise, family farming interests also perceived the “healthiness” of their foods in terms of freshness. “Children had powdered milk before and now they can drink fresh goats’ milk”, said one official concerned with family farming. According to another, “Before, the schools served artificial juices. Now we have *umbu* juice being served in schools. Many times, a child had never had *umbu* juice”^r^. Others mentioned the relationship of children with their food. “We want food to which children are connected” said one.

The nutritionists interviewed agreed that family farms were able to provide the “basic” foods needed in PNAE^s^. But they did not think that food was “healthy” just because it came directly from family farms. “The supply of products from family farms is not necessarily healthy”, said one nutritionist, who is highly experienced in working with family farmers, “healthy eating does not happen just because we buy from the family farm”. According to another:

We are taking the middle man out of this process and the food is cheaper. But this does not mean that family farm products are necessarily “healthy foods” and that a healthy diet cannot be bought from the food industry. The industry often buys the food from the family farmer and then sells it. Is a lettuce not healthy because it is sold by industry rather than direct from a family farmer? Can’t I buy fresh milk from industry? Could I say that this is not healthy just because it comes from industry?

As such, the “strong voice” of the nutritionists in the CONSEA working group advocated for another Article in the law – the requirement that a nutritionist design the menus for the PNAE. This was, according to one of the nutritionist working group members, one of “the easiest points of consensus in the group”. In addition, the implementing resolution for Law 11,947 (approved some months later) included specific nutritional standards for school meals.

It is clear, then, that to improve nutritional outcomes was not the primary goal of Article 14. Indeed, its stated aim was to support local economic development, not to improve nutrition^t^. Was this, then as put by one informant, a failure of intersectorality? Was it a missed opportunity for nutrition to be a more explicit goal alongside economic development for family farmers?

Arguably, no, because, politically, it served the nutrition interest *not* to focus on the nutrition technicalities – which were anyway within their sole area of expertise, and not an interest of coalition partners, and, moreover, could have derailed the process^u^ – but on a common goal. According to a food security advocate: “Some people cared about nutrition and health; others cared about family farming; others about economic development and poverty. But for everyone, changing the PNAE was a goal”.

There is an important political lesson here. The nutritionists involved in the policy process that led to Article 14 were not a powerful political force, nor part of a separate advocacy coalition. But they had their own objective: an enlarged PNAE serving better food. While technically they did not believe it was essential to source food directly from family farmers for it to be healthy in a nutritional sense, politically they saw that participating in coalitions with others was a means of gaining support for overall improvements and moving the program in the general direction they wanted to go – more basic foods like fresh fruits and vegetables etc. Important as it was for nutritionists to be present and have a clear nutrition objective, not focusing on that goal *as* the foundation of the coalition was politically advantageous.

Also critical here was that family farming is a subsector more powerful than nutrition/health interests and holds greater political sway. According to one informant, “politically, it was a good tactic. It is hard for the politician to say: No, I am against family farming”. In forging alliance with family farmers, then, the nutrition and health interest was recognizing the political opportunity presented by family farming as a newly powerful force in government, reflecting on the importance of “building on positive factors in the policy environment to ensure political support” for intersectorality^15^.

It is worth noting that in calling for more intersectoral action, the international nutrition community has called for “nutrition-focused development”, which “seeks to promote adequate nutrition as the goal of national development policies in agriculture, food supply”^22^. Yet, the lesson here is that when it comes to the important process of building a coalition with other sectors, nutrition does not have to be *the* explicit goal. More important is the political process of identifying and participating in a strong coalition that is able to cause change in the right direction, focused – and fighting for – on a common goal into which nutrition and health can fit.

### Lesson Three: Positioning Nutrition and Health Goals as a Solution that Meets the Interests of Other Sectors

There is another related lesson here: Article 14 explicitly met the interests of family farmers. The attractions were clear – 3.1 billion reais spent per year on food, 30.0% of which could now be allocated to family farmers without the need for more money. For them, it was a matter of “redirecting existing resources rather than having to lobby for new resources”. “You have to buy the basic basket. Good! Why not buying it from the family farming?” said one. The inclusion of Article 14 – and only that – was the reason that family farming interests came on board to support the bill. As one key informant said, “it was like someone from the family farming community read the draft bill and was shocked and then took it on as their cause […] they bought this as their project and started to support it”.

The important lesson here is that the incentive for intersectorality does not come from a problem, but from a solution: Article 14 met the interests of a more powerful sector – family farmers. For them, it was this incentive – new markets and income generation – that was needed to stimulate intersectoral action.

### Lesson Four: Obtaining Evidence that the Intersectoral Approach Can Work

Article 14 was not the first initiative in Brazil linking family farmers with markets. Most notable was the Food Acquisition Program (PAA) established in 2003 (see Lesson One)^7^ ^,^ ^23^. The Executive Steering Group established for the PAA was inter-governmental (see Lesson 1), and perceived a link with schools meals as a “natural progression” from the PAA.

Indeed, the PAA was said to be the “father and grandfather” of Article 14. It was considered crucial because it provided evidence. According to one informant, “the safe five years background of the PAA made us believe that buying from family farms works… it established the idea in practice and gave us confidence it could be implemented as public policy”. For the FNDE – which were reported to be highly skeptical early on that sourcing directed from family farms was legal and possible – “the PAA gave the empirical evidence that it is possible to buy from family farmers,” notably that pricing and procurement mechanism could work and family farmers could supply sufficient food. As stated by an informant from CONAB, “the PAA paved the way for everything because it demonstrated that family farmers were able to produce food in sufficient quality, diversity and quantity”. It managed to “break the myth” that this was impossible. Thus, as said by an MDA official, “without the PAA experience we would unlikely have managed to regulate and maintain the resolution”.

Importantly, this evidence was crucial in defeating the opposition to Article 14.

The argument of opponents – who were considered supporters of the senator in opposition – was said to be consistent: that family farmers could not provide enough food for the PNAE, they could not provide it on a regular basis, and did not have the logistical infrastructure required to do so. These arguments held some sway over members of the Congress, some of whom introduced amendments to lower the 30.0% purchase to 10.0% or 5.0%. Indeed, according to the coordinator of PNAE, the most difficult aspect of Article 14 was “to persuade the members of the Congress that there was enough production for at least 30.0%”. It was proof from the PAA, combined with data from the family farming sector, that “there was no way it could be questioned”. The outsourcing article had to be extinguished, but Article 14 remained.

Still, it was impossible to change the bill into law without including the “exceptions” that permitted municipalities to not implement the 30.0% clause under certain circumstances. This was reportedly a huge discussion. The social movements were rigidly opposed because they were afraid that the exception would invalidate the rules. Yet the FNDE and the deputy leader of the Parliamentary Front drafted the exceptions as a compromise because they satisfied the “concerns of the mayors that they would not have to take responsibility if the farmer could not produce”. The inclusion of the exceptions, thus, “helped build consensus in the chamber of deputies and in the senate” and the bill would reportedly “not have been approved without them”.

### Lesson Five: Not Being Afraid of Bold Ideas When Working with Other Sectors

The inclusion of Article 14 in a law was, according to one informant, “outrageous in its boldness”, which led to, in the words of another, “a law of great daring”. What was so important here was that Article 14 was appealing as an idea. It not only had the political appeal of supporting family farmers and economic development, but (even though “health” was an unimportant part of the discussion) “it was an important political force in the minds of the population, the social imagination and the enhancement of self-esteem of the farmers because it will nourish the children”. As stated by an informant from the farming movement, “It is a very nice idea, right? Who is going to be against it? If you say that we produce healthy food by the Brazilian people for the Brazilian people, people are delighted. At last, we will eat a good meal. As a result, the law has a very good acceptance”.

What might sound like an “outrageous” idea in one sector may have much broader appeal elsewhere. Principles, language, assumptions, and approaches change when working with other sectors: they can be exploited for mutual advantage.

## CONCLUSIONS

This study of policy processes shows how a convergence of factors merged to open up a policy window for Article 14 to be drafted and changed into law^12^, and provides lessons on how to advance nutrition and public health goals in the agricultural sector and others.

After six years of the publication of the Article 14 of Law 11.947, huge efforts have taken place to implement it despite significant challenges. In 2012, 81.0% of municipalities bought directly from family farmers and 50.0% met the minimum purchase of 30.0%^5^. Although the goals have not been fully met, the purchase of family farmers is now an established practice in the PNAE^19^ ^,^ ^24^.

But an important question remains. Will the implementation of Article 14 actually have any impact on its potential outcomes, such as on local economic development, the strengthening family agriculture, and, in particular, the quality of school meals and the diets of school children? More evaluative studies are necessary, and their results will show whether the battle for Article 14 proved to be just a political tool to win gains for economic development and food security or whether it will also bring gains for the diet quality and health of the millions of people receiving public education in Brazil.

## Footnotes

a Brasil. Lei nº 11.947, de 16 de junho de 2009. Dispõe sobre o atendimento da alimentação escolar e do Programa Dinheiro Direto na Escola aos alunos de educação básica. *Diario Oficial Uniao.* 17 jun 2009;Seção 1.

b A term unique to Brazil referring to isolated communities composed of escaped slaves, originally formed many decades ago.

c Ministério da Educação. Fundo Nacional de Desenvolvimento da Educação. Programas: Alimentação Escolar (PNAE). Brasília (DF): Fundo Nacional de Desenvolvimento da Educação; 2015 [cited 2015 Jun 10]. Available from: http://www.fnde.gov.br/programas/alimentacao-escolar

d The blueprint for *Fome Zero* was written in 2001 when Lula was director of *Instituto Cidadania* (political group that was against the ideals proposed by the president of Brazil at the time, Fernando Collor de Melo, and which started developing several political activities in order to better understand the geopolitical, social, and economic scenario of Brazil after the president’s impeachment) while in opposition. The first year of implementation was unsuccessful.

e The first CONSEA was established by the interim government of Itamar Franco, but it was dissolved in 1995 when President Fernando Henrique Cardoso came to power. In addition to CONSEA, the *Ministério Extraordinário de Segurança Alimentar e Combate à Fome* (MESA – Brazilian Extraordinary Ministry for Food Security and Fight Against Hunger), was created to lead *Fome Zero* Program; MESA later became the *Ministério do Desenvolvimento Social e Combate à Fome* (MDS – Brazilian Ministry of Social Development and Fight Against Hunger).

f CONSEA comprises around 40 civil society representatives, 19 government ministers and representatives of the Federal Government, and around 30 invited observers, with a president selected from civil society.

g Brasil. Lei nº 11.346, de 15 de setembro de 2006. Cria o Sistema Nacional de Segurança Alimentar e Nutricional – SISAN com vistas em assegurar o direito humano à alimentação adequada e dá outras providências. *Diario Oficial Uniao.* 15 set 2006.

h The original *Fome Zero* blueprint had noted that the provision of food from local farmers would be one means of increasing the freshness of food in the program. The second *Conferência Nacional de Segurança Alimentar e Nutricional* (CNSAN – National Conference on Food and Nutrition Security), in 2004, identified both family farming and healthy eating – and their relation by PNAE – as priority area. Two years later, the *Lei Orgânica de Segurança Alimentar e Nutricional* (LOSAN – Lei nº 11.346, de 15 de setembro de 2006 Law of Food and Nutrition Security), which enshrined the “right to adequate food”, called for the “expansion of conditions of access to food by production, in particular traditional and family agriculture”.

i There are two ministries of agriculture in Brazil, the other being the *Ministério da Agricultura, Pecuária e Abastecimento* (MAPA – Brazilian Ministry of Agriculture, Livestock, and Food Supply), which takes care of agribusiness interests in Brazil. The *Companhia Nacional de Abastecimento* (CONAB – National Food Supply Company) is an agency of MAPA, but also has some responsibility for family farmers (Diretoria de Política Agrícola e Informações - DIPAI).

j PAA became the first program unrequired to follow the competitive bidding process established in the procurement law of 1993, which had generally favored procurement from larger, capital intensive entities. Rather, the program adopted a reference pricing mechanism based on an “average price” rather than on a “minimum price”, which facilitated the participation of family farmers while remaining a competitive process.

k “Family farming” was defined in Law 11,326 in 2006, using a definition from PRONAF established in 1995.

l CONSEA. II Conferência Nacional de Segurança Alimentar e Nutricional. A construção da Política Nacional de Segurança Alimentar e Nutricional. Relatório final; 2014 [cited 2015 Oct 24]. Available from: http://bvsms.saude.gov.br/bvs/publicacoes/II_Conferencia_2versao.pdf

m
*Casa Civil* (the Civil House) is a department in the executive part of the government responsible for reviewing bills for legal adequacy, political expediency, and financial feasibility. In this case, the bill remained in *Casa Civil* for many months. During the process, the wording of the bill was changed, eliminating some aspects but maintaining Article 14.

n Executive Steering groups included representatives of MDA, MAPA (including CONAB), MDS, the Brazilian Ministry of Finance and Planning, and later, the Brazilian Ministry of Education.

o In 2007, the III CNSAN, attended by about 1,800 people, discussed the potential link between family farming and PNAE. President Lula attended it, and in his speech he stated his support for the draft bill. As a result, the Parliamentary Front began to engage more comprehensively in support of the emerging bill that had now been drafted by CONSEA.

p This redrafting involved the conversion of the bill to a Provisional Measure (MP) in order to prevent the bill from being dropped because of time limits. The MP was tabled and approved by the president on January 28, 2009.

q The origin of inadequate quality food in the PNAE was consistently attributed by key informants to its original structure. The program had been centralized, involving the distribution of pre-packaged (often dehydrated) foods from central locations to all regions of Brazil, focusing on providing calories, not quality diets. Although the food quality improved significantly after food purchasing power was transferred to the municipalities in 1994 (which were required to favor regional products *in natura* in their menus, the “cookies from Sao Paulo”, as informants refer to the food), the food continued to be “exported” to states thousands of miles away.

r For a few of the informants, the issue of agrochemicals also featured strongly. “When we think of healthy, we are not just thinking of nutrition”, said one, “but whether it is produced sustainably, without agrochemicals or genetically modified organisms (GMOs)”.

s COTAN had come to this conclusion after reviewing the menus side-by-side with data on family farming production.

t Overall, despite the recognition of the problem and actions taken by the Brazilian Ministry of Health, it concerns about “healthy eating” and obesity failed to gain any political traction during Lula’s presidency.

u For example, the definition “basic foods” included in an earlier draft was removed during the redrafting period and the conversion to the MP, despite its importance in the discourse, because there was no common consent about what its interpretation from a nutritional perspective. At an earlier stage, has also been considerable difficulty with discussions within CONSEA about what was really meant by “healthy food”.

## References

[1] Breton E, De Leeuw E. (2011). Theories of the policy process in health promotion research: a review. Health Promot Int..

[2] Bundy D, Burbano C, Grosh M, Gelli A, Jukes M, Drake L (2009). Rethinking school feeding: social safety nets, child development, and the education sector.

[3] Burlandy L (2009). Construction of the food and nutrition security policy in Brazil: strategies and challenges in the promotion of intersectorality at the federal government level. Cien Saude Colet..

[4] Buse K, Dickinson C, Gilson L, Murray SF (2009). How can the analysis of power and process in policy-making improve health outcomes?. World Hosp Health Serv..

[5] Câmara Interministerial de Segurança Alimentar e Nutricional (2013). Balanço do Plano Nacional de Segurança Alimentar e Nutricional: PLANSAN 2012/2015.

[6] Canon G (2004). Why the Bush administration and the global sugar industry are determined to demolish the 2004 WHO global strategy on diet, physical activity and health. Public Health Nutr..

[7] Chmielewska D, Souza D (2010). Market alternatives for smallholder farmers in food security initiatives: lessons from the Brazilian Food Acquisition Programme.

[8] Espejo F, Burbano C, Galliano E (2009). Home-grown school feeding: a framework for action.

[9] Food and Agriculture Organization of the United Nations (2004). Economic and social development as the basis for FAO actions in Latin America and the Caribbean.

[10] Geneau R, Stuckler D, Stachenko S, McKee M, Ebrahim S, Basu S (2010). Raising the priority of preventing chronic diseases: a political process. Lancet.

[11] Guanziroli CE, Cardim SECS (2000). Novo retrato da agricultura familiar: o Brasil redescoberto.

[12] Kingdon JW (1984). Agendas, alternatives and public policies.

[13] (2015). Leverhulme Centre for Integrative Research on Agriculture and Health (LCIRAH).

[14] Morgan K, Sonnino R (2008). The school food revolution: public food & the challenge of sustainable development.

[15] Public Health Agency of Canada (2007). Crossing sectors: experiences in intersectoral action, public policy and health.

[16] Sabatier PA, Jenkins-Smith HC (1993). Policy Change and learning: advocacy coalition approach (theoretical lenses on public policy).

[17] Santos MJ (2001). Projeto alternativo de desenvolvimento rural sustentável. Estud Av..

[18] Santos LMP, Santos SMC, Santana LAA, Henrique FCS, Mazza RPD, Santos LAS (2007). Avaliação de políticas públicas de segurança alimentar e combate à fome no período 1995-2002. 4 – Programa National de Alimentação Escolar. Cad Saude Publica.

[19] Saraiva EB, Silva APF, Sousa AA, Cerqueira GF, Santos CM, Toral N (2013). Panorama da compra de alimentos da agricultura familiar para o Programa Nacional de Alimentação Escolar. Cienc Saude Coletiva.

[20] Silva JB (2015). Programa Nacional de Fortalecimento da Agricultura Familiar – PRONAF.

[21] Todd ECD, Narrod C (2006). Understanding the links between agriculture and health: agriculture, food safety, and foodborne diseases.

[22] United Nations Standing Committee on Nutrition (2010). A road map for scaling-up nutrition (SUN).

[23] Vaitsman J, Paes-Sousa R (2007). Evaluation of MDS policies and programs: results.

[24] Villar BS, Schwartzman F, Januario BL, Ramos JF (2013). Situation of the municipalities of São Paulo state in relation to the purchase of products directly from family farms for the National School Feeding Program (PNAE). Rev Bras Epidemiol..

[25] World Bank (2006). Repositioning Nutrition as central to development: a strategy for large-scale action.

[26] World Health Organization (2004). Global strategy on diet, physical activity and health.

